# Supplementary figures and images for: Transcriptomic Analysis Reveals Key Pathways Influenced by HIV-2 Vpx
Source: Int J Mol Sci. 2025 Apr 8;26(8):3460. doi: 10.3390/ijms26083460 (PMC12026760; doi:10.3390/ijms26083460)

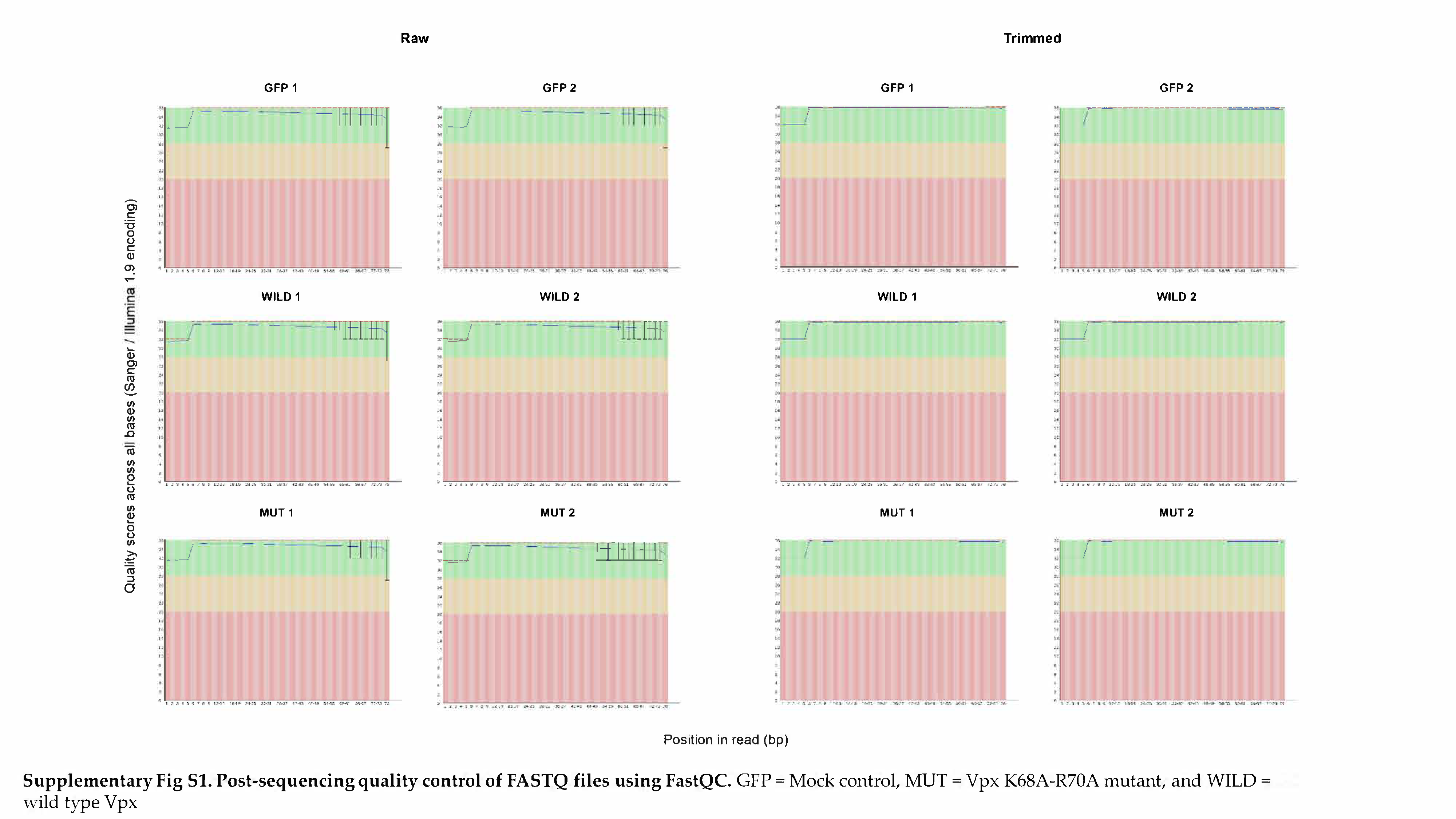

Supplement: Supplementary file 1 [file ijms-26-03460-s001.zip › SF1.png]

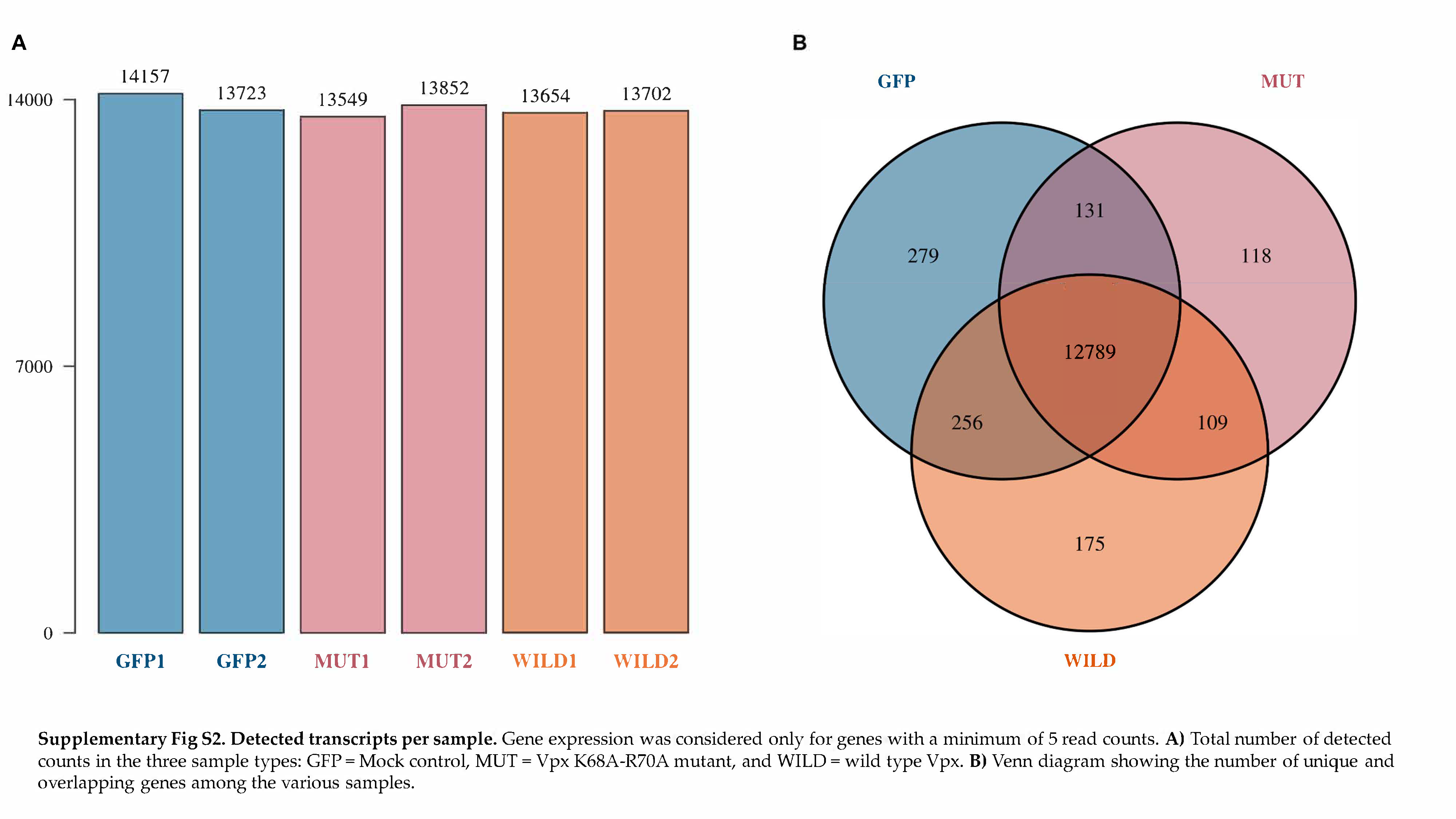

Supplement: Supplementary file 1 [file ijms-26-03460-s001.zip › SF2.png]

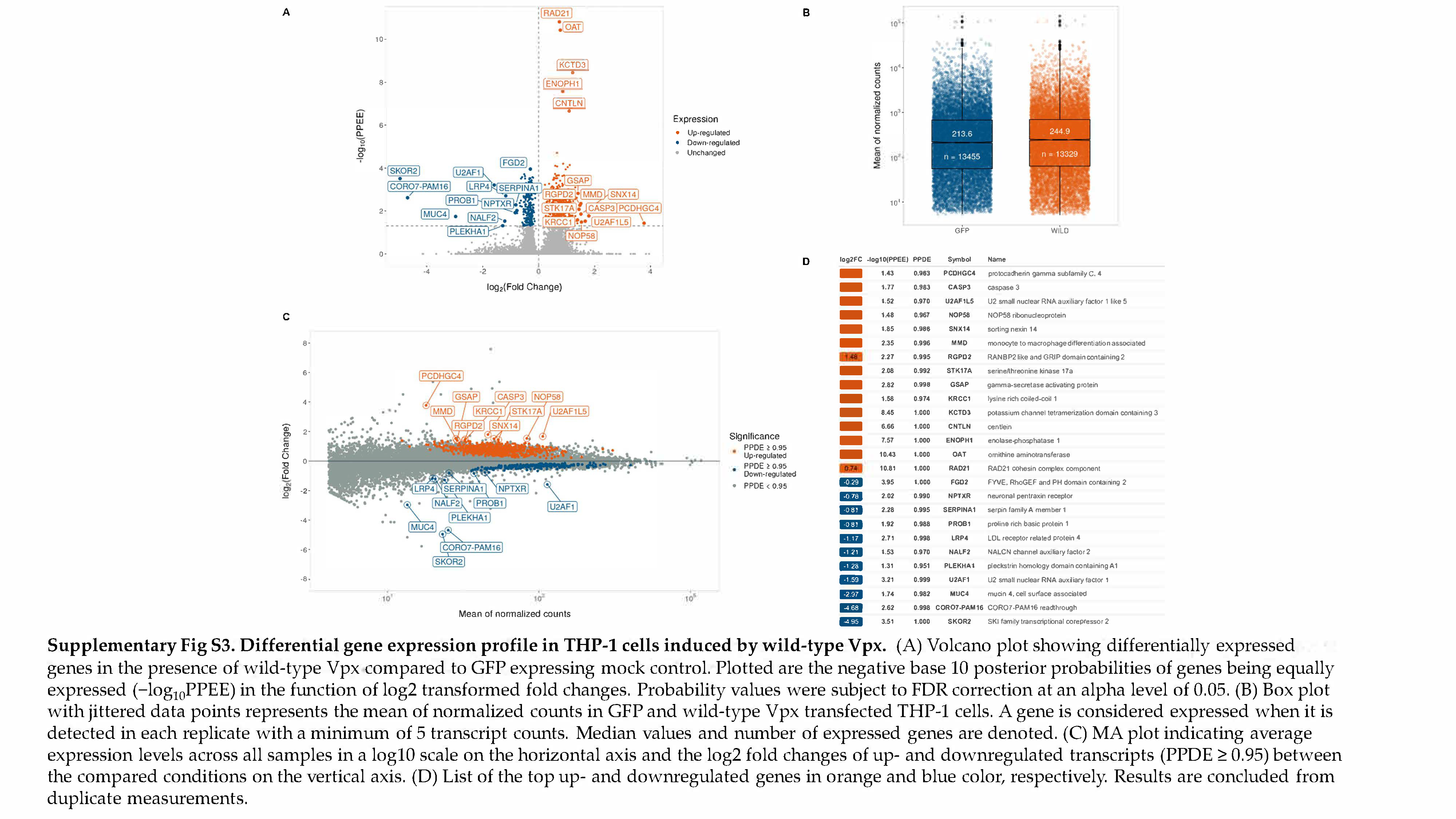

Supplement: Supplementary file 1 [file ijms-26-03460-s001.zip › SF3.png]

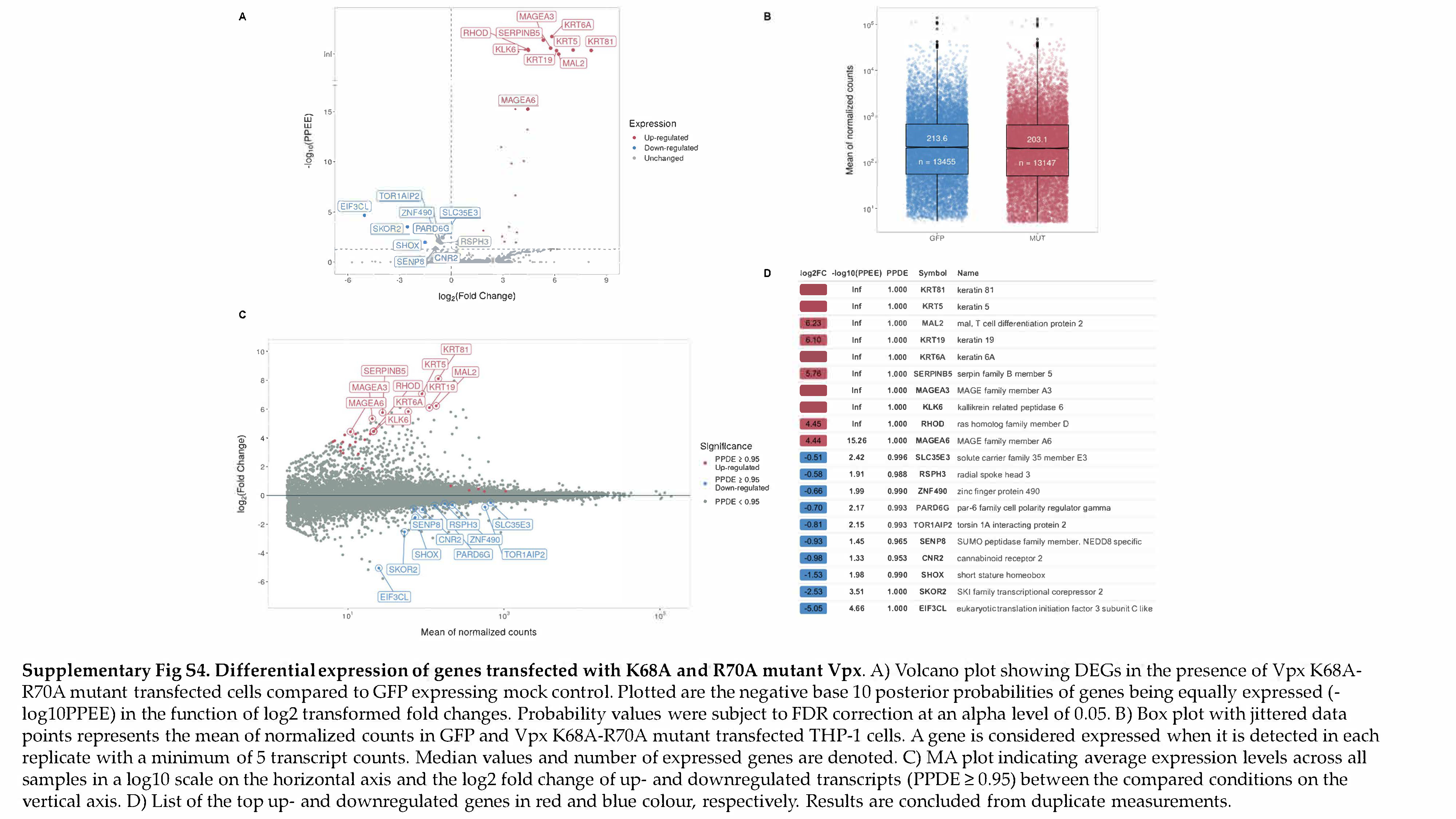

Supplement: Supplementary file 1 [file ijms-26-03460-s001.zip › SF4.png]

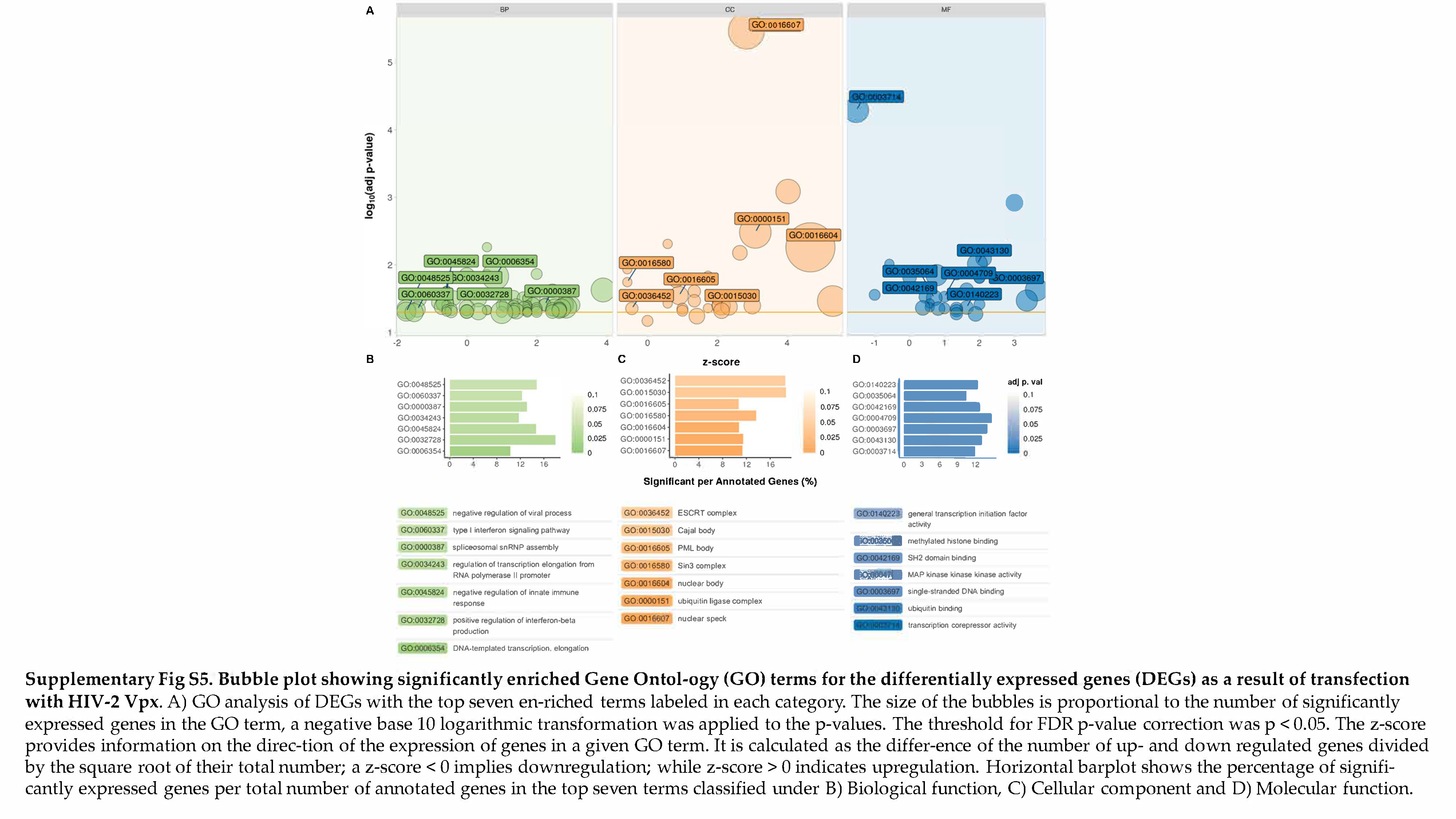

Supplement: Supplementary file 1 [file ijms-26-03460-s001.zip › SF5.png]

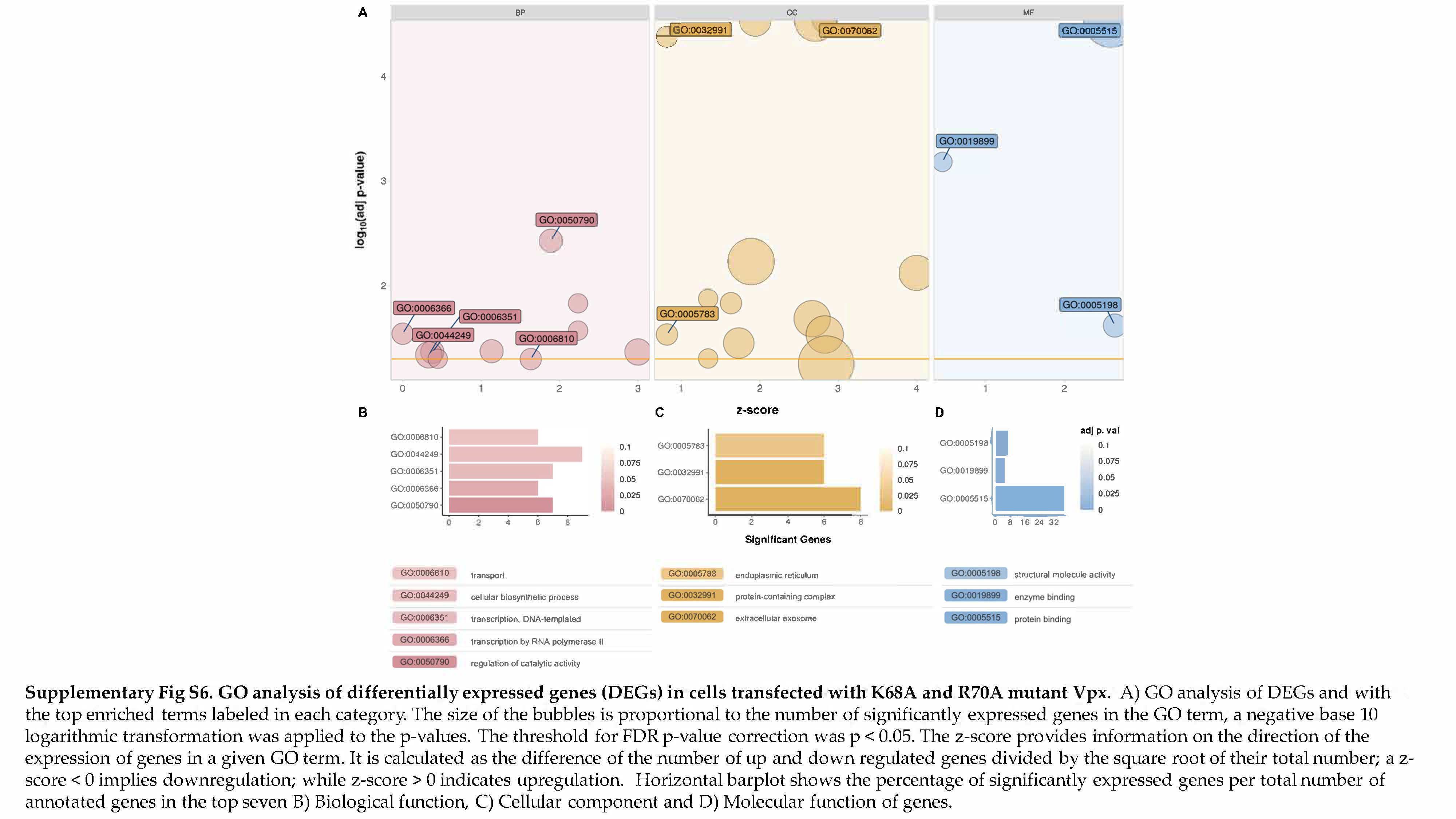

Supplement: Supplementary file 1 [file ijms-26-03460-s001.zip › SF6.png]

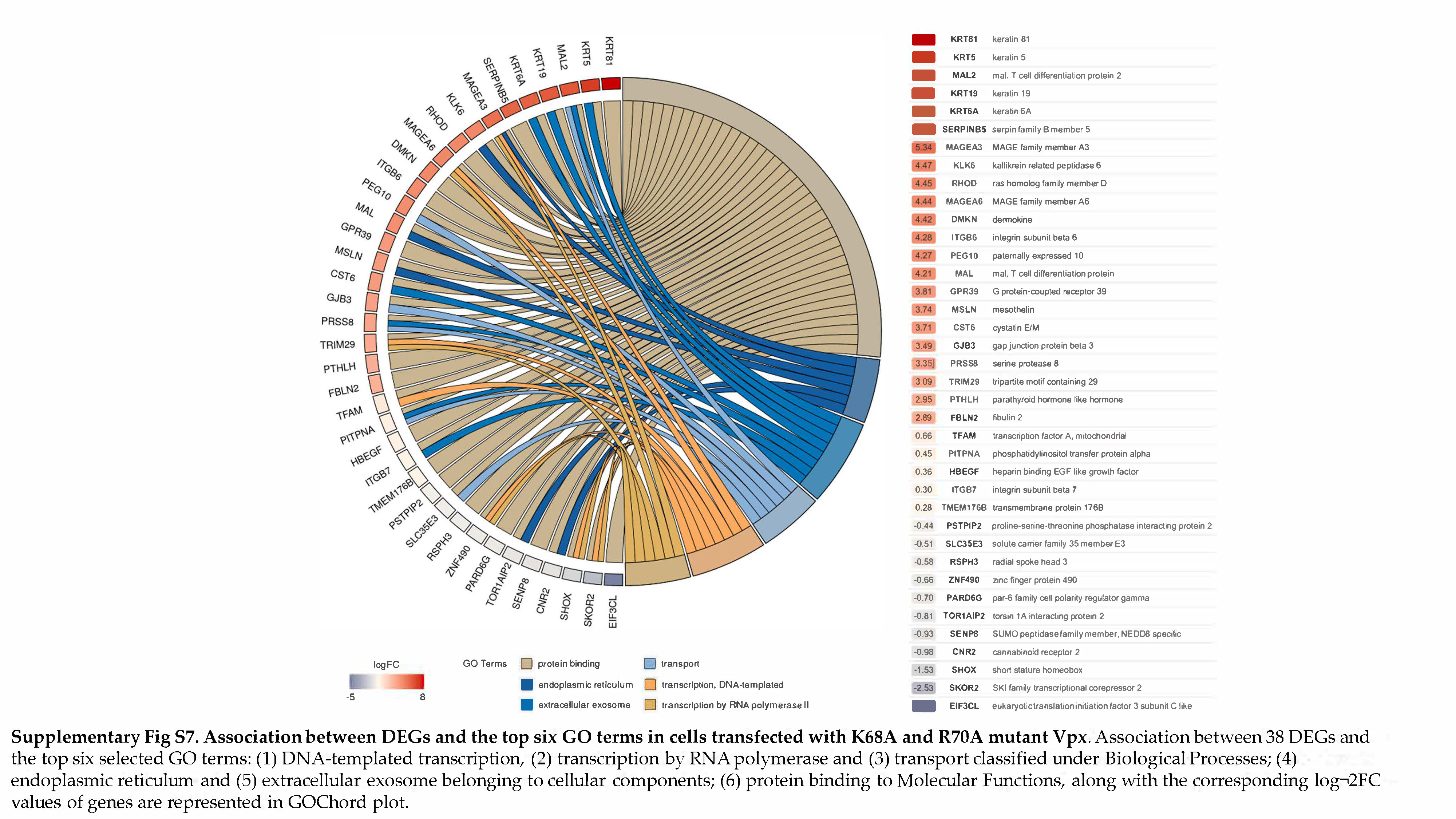

Supplement: Supplementary file 1 [file ijms-26-03460-s001.zip › SF7.png]
